# Thermally-induced drift of A-site cations at solid–solid interface in physically paired lead halide perovskites

**DOI:** 10.1038/s41598-022-14452-y

**Published:** 2022-06-17

**Authors:** Daniele T. Cuzzupè, Feray Ünlü, Khan Lê, Robin Bernhardt, Michael Wilhelm, Matthias Grosch, Rene Weißing, Thomas Fischer, Paul H. M. van Loosdrecht, Sanjay Mathur

**Affiliations:** 1 Chemistry Department, Institute of Inorganic Chemistry, University of Cologne, Greinstr. 6, 50939 Cologne, Germany; 2 Institute of Physics 2, University of Cologne, Zülpicher Str. 77, 50937 Cologne, Germany; 3 Present Address: Department of Physics, University of Konstanz, 78457 Constance, Germany

**Keywords:** Solar cells, Optical materials

## Abstract

The promise of hybrid organic–inorganic halide perovskite solar cells rests on their exceptional power conversion efficiency routinely exceeding 25% in laboratory scale devices. While the migration of halide ions in perovskite thin films has been extensively investigated, the understanding of cation diffusion remains elusive. In this study, a thermal migration of A‑site cations at the solid–solid interface, formed by two physically paired MAPbI_3_ and FAPbI_3_ perovskite thin films casted on FTO, is demonstrated through continuous annealing at comparably low temperature (100 °C). Diffusion of methylammonium (CH_3_NH_3_^+^, MA^+^) cations into the low‑symmetry yellow δ‑FAPbI_3_ phase triggers a transition from the yellow (δ) to black (α) phase evident in the distinctive color change and verified by shifts in absorption bands and X‑ray diffraction patterns. Intermixing of the A‑site cations MA^+^ and FA^+^ (CH(NH_2_)_2_^+^) occurred for both systems, α‑MAPbI_3_/δ‑FAPbI_3_ and α‑MAPbI_3_/α‑FAPbI_3_. The structural and compositional changes in both cases support a thermally activated ion drift unambiguously demonstrated through changes in the absorption and X-ray photoelectron spectra. Moreover, the physical contact annealing (PCA) leads to healing of defects and pinholes in α‑MAPbI_3_ thin films, which was correlated to longer recombination lifetimes in mixed MA_x_FA_1−x_PbI_3_ thin films obtained after PCA and probed by ultrafast transient absorption spectroscopy.

## Introduction

Advancing the initial implementation of organic–inorganic lead halide perovskites (APbX_3_) in solid-state planar devices^1^, predominantly with A-site cations such as methylammonium (CH_3_NH_3_^+^_,_ MA^+^) or formamidinium (CH(NH_2_)_2_^+^, FA^+^)^2,3^, the efficiency of the perovskite solar cells (PSCs) has surged rapidly from about 4% to exceed 25% in less than a decade^4,5^. To this end, exhaustive efforts have been undertaken to optimize the stability and efficiency of the photovoltaic devices illustrated in conclusive data on the influence of the variation of the perovskite composition, investigation of charge-transport layers and modelling of multi-material interfaces^6^. The necessity of optimizing the perovskite devices in terms of operational durability and currently unresolved materials challenges are detailed in recent reviews on PSCs^7^. The promising device-related properties of MAPbI_3_ include narrow direct band gap (1.55 eV), high absorption coefficient (α ∼ 10^5^ cm^−1^)^8^, low exciton binding energy (ca. 16 meV) ensuring charge carrier generation at low temperatures^9^, long-ranged diffusive transport (up to 1 μm) and high charge carrier mobilities (1–100 cm^2^/Vs)^10,11^. In combination with these advantageous functional properties, the band gap tunability through compositional control and facile solution-based processing of high-quality photoactive MAPbI_3_ layers had fueled the technology readiness levels of PSCs evident in large scale demonstrators^12–14^.

Despite significant progress in the fundamental understanding of the photo-physical properties of hybrid perovskites, the progress is confronted with the limited structural stability of the photoabsorber materials and their interfacial reactivity in multi-layered planar devices. For example, the device applications of well-suited MAPbI_3_ are challenged by a facile phase transition (tetragonal-to-cubic) at low temperature (57 °C) that falls within the operational temperature. This indicates the thermal instability of organic–inorganic lead halide perovskite, which triggers high sensitivity towards moisture ultimately leading to the degradation of the perovskite structure^15–17^. To improve phase stability while maintaining PSC-relevant properties, organic cations such as formamidinium and guanidinium (C(NH_2_)_3_^+^ or GA^+^) as well as inorganic cesium cation (Cs^+^) were tested in mixed-cation perovskites to stabilize the perovskite structure^18–20^. The use of formamidinium as A-site cation in lead halide perovskite solar cells has gradually increased since the early reports in 2014 mainly due to its structure-stabilizing effect. In comparison to MA^+^, FA^+^ has a larger ionic radius of 253 pm and the resulting FAPbI_3_ perovskite shows lower band gap (1.48 eV) in comparison to MAPbI_3_^21^, that makes FAPbI_3_ a more promising candidate for extending absorption in the longer wavelengths range^3^. Furthermore, it is more stable than MAPbI_3_ at higher temperatures (150 °C), even though like the methylammonium analog it is moisture-sensitive and tends to degrade to PbI_2_ in humid environment^3^. The main challenge of FAPbI_3_ is the spontaneous transition below 150 °C from the photoactive black α-phase to the non-perovskite yellow δ-phase, which does not exhibit photovoltaic properties^22^. The first approach of mixing multiple A-site cations was aimed at combining the stability of the black phase of MAPbI_3_, with the more favorable band gap of FAPbI_3_. In fact, the presence of MAPbI_3_ was shown to suppress the formation of the parasitic yellow δ-phase of FAPbI_3_. Moreover, the red-shift in the band gap led to an increase in the photocurrent with no damaging effect on the voltage^23^.

The high structural tolerance of the perovskite framework is responsible for the multiple phase transitions that alter the intrinsic properties observed for instance in abnormal hysteresis between forward and reverse current–voltage (J–V) scans during device operation^23^. A possible explanation for this phenomenon is the migration and accumulation of halide ions at the electrodes^24,25^. A-site cation engineering and halide mixing have been crucial in the improvement of the power conversion efficiencies (PCE) of PSCs, where multi-cation perovskites have been demonstrated to increase the phase stability and halide mixing allows band gap tuning^13^. Ion migration in mixed-halide perovskites represents a prominent reason for the observed fluctuations in charge transport and dynamics^26^. Predominantly, the ion migration in metal halide perovskites occurs through vacancy-mediated processes, as the interstitial migration is less favorable in densely packed perovskite structure^27^. The vacancy-mediated diffusive mechanism was supported by *Eames *et al. who calculated the activation energies for the vacancy-mediated migration of MA^+^ (0.84 eV), Pb^2+^ (2.31 eV), and I^−^ (0.58 eV) ions^27^ indicative of a virtually immobile Pb sublattice. Within this static framework, the iodide and MA^+^ ions can migrate, however, the calculated diffusion coefficient of MA^+^ was found to be four orders of magnitude lower than that of I^−^ anions^27^. Ion migration in lead halide perovskite layers has also been associated with light soaking and voltage bias treatment. Interestingly, *Xiao *et al. reported on a switchable photovoltaic effect in which the polarity of simple metal/perovskite/metal devices could be reversibly tuned via a small electric field and attributed the effect to ion and charge carrier drift upon the application of the voltage bias^28^. Furthermore, it was shown that halide ions in mixed-halide lead perovskite thin films redistribute upon light illumination, resulting in undesired phase segregation, nonetheless, the mixed-halide composition could be recovered upon storing the films in the dark^29^. Considerably less attention is paid to the migration of A-site cations, partially due to the complexity of tracing simple and small organic molecules with analytical techniques. However, in an extensive work by *Domanski *et al. supported by theoretical models, it is demonstrated that cations in lead halide perovskite solar cells do indeed migrate within a slow timescale, at the expense of the PV performances of the devices^30^.

To deepen the understanding of ion migration phenomena in lead halide perovskites, *Elmelund *et al*.* investigated the halide solid-to-solid diffusion between two different perovskite thin films with a different composition (MAPbI_3_ and MAPbBr_3_) when the two films were physically paired under continuous annealing^31^. Herein, we adapted the concept to the investigation of solid-to-solid A-site cation diffusion between MAPbI_3_ and FAPbI_3_ thin films and focused on solid-to-solid cationic diffusion to elaborate the effects of cation migration on structural and consequently photo-physical properties in the perovskite bilayers in contact.

## Results and discussion

**Figure 1:**
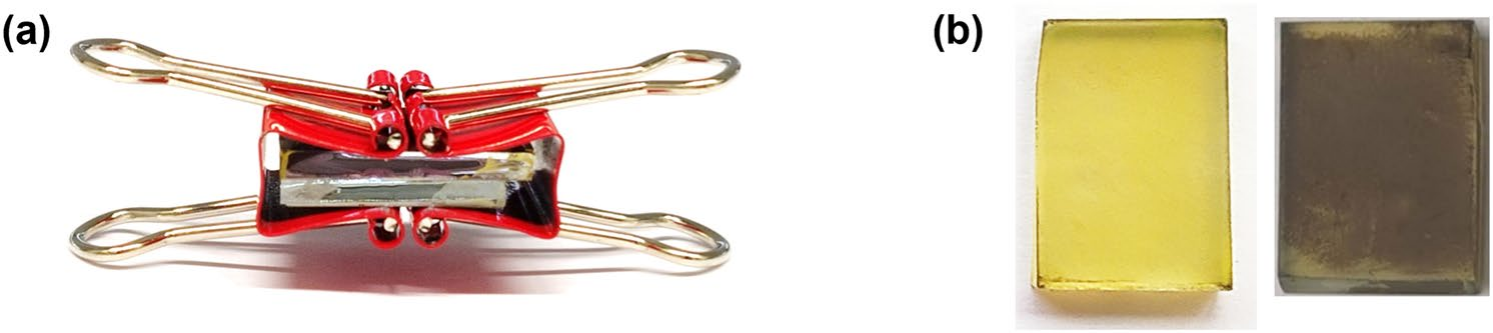
(**a**) Perovskite-coated substrate in physical contact for PCA. (**b**) color change of the FAPbI_3_ sample from yellow (as-prepared) to grey (after 20 h PCA).

The investigated thin films of α‑MAPbI_3_ and δ‑FAPbI_3_ were fabricated via spin-coating of precursor solutions onto pre-cleaned fluorine-doped tin oxide (FTO) substrates in a nitrogen atmosphere under controlled humidity. After short annealing treatments to remove residual solvents, a pair of substrates α‑MAPbI_3_/δ‑FAPbI_3_ were physically paired in face-to-face fashion (Fig. 1a) and annealed for 20 to 80 h at 100 °C. We have termed this process ‘physical contact annealing’, hereinafter referred to as ‘PCA’.

**Figure 2:**
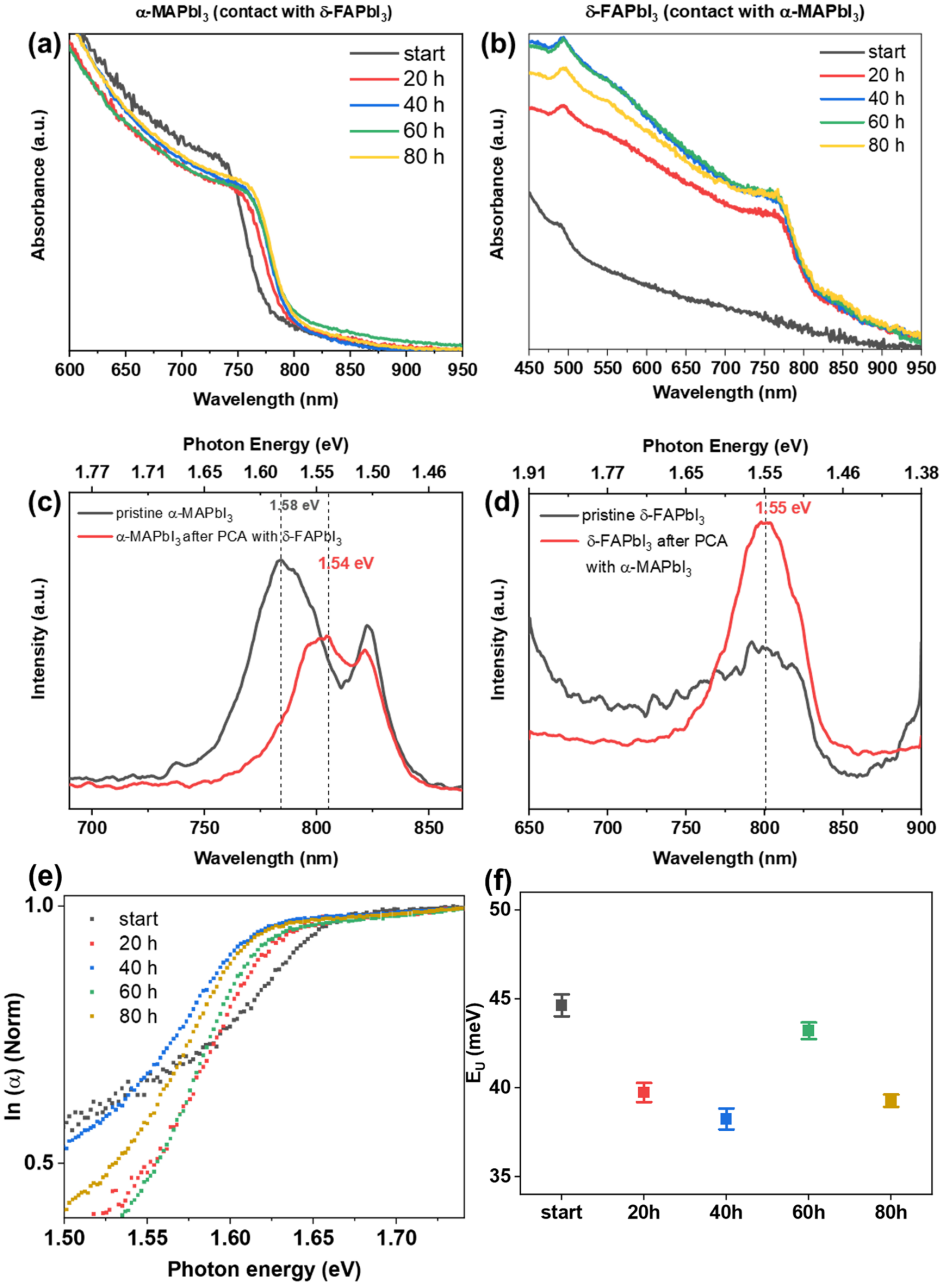
UV–vis spectra of MAPbI_3_ (**a**) and FAPbI_3_ (**b**) thin films after different times of physical contact annealing (PCA) at 100 °C. PL emission of (**c**) MAPbI_3_ before and after 80 h PCA and (**d**) FAPbI_3_ before and after 80 h PCA. (**e**) Normalized plots used for the calculation of the estimated Urbach energy. (**f**) Calculated *E*_U_ values.

Figure 2a,b present the UV–vis spectra of α‑MAPbI_3_ and δ‑FAPbI_3_ samples, respectively, recorded over 80 h. The progressive absorption band edge shift from 782 nm towards higher wavelength (804 nm) for MAPbI_3_ indicated a change in the estimated band gap (from 1.60 to 1.57 eV, Fig. S1). Interestingly, FAPbI_3_ exhibited an absorption onset at 830 nm supported by a color change from yellow to black after 20 h PCA (Fig. 1b), suggesting a phase change. We found a corroboration of these preliminary observations in the photoluminescence (PL) spectra of the thin films. For MAPbI_3_, the main PL emission band shifted from 1.58 eV in the pristine thin film to 1.55 eV after PCA (Fig. 2c). For FAPbI_3_, the pristine material did not show an emission band in the investigated region, whereas one single band at 1.55 eV was observed after PCA treatment (Fig. 2d). These findings suggested that upon physical contact annealing, MA^+^ and FA^+^ cations gradually migrate into the opposite thin film to yield intermixed MA_1−x_FA_x_PbI_3_ and MA_x_FA_1−x_PbI_3_ compositions, respectively. The UV–vis absorption spectra can also be used to estimate the Urbach energy (*E*_U_) of the material over the annealing time. The used approximations are explained in Note 1 (Supporting Information) and the fitted plots are shown in Fig. S3. Figure 2e displays the normalized ln (α) plotted against the photon energy. In Fig. 2f, the calculated Urbach energies are shown. Interestingly, we find that in the first 40 h PCA, *E*_U_ decreases from 44.5 to 38.5 meV. Usually, smaller E_U_ values are associated with a less defective structure of the materials, which is often correlated to improved electronic properties^32^. The oscillation of the E_U_ values at 60–80 h PCA could indicate an interplay between PCA-induced enhancement of the structural quality and degradation driven by ambient conditions.

**Figure 3:**
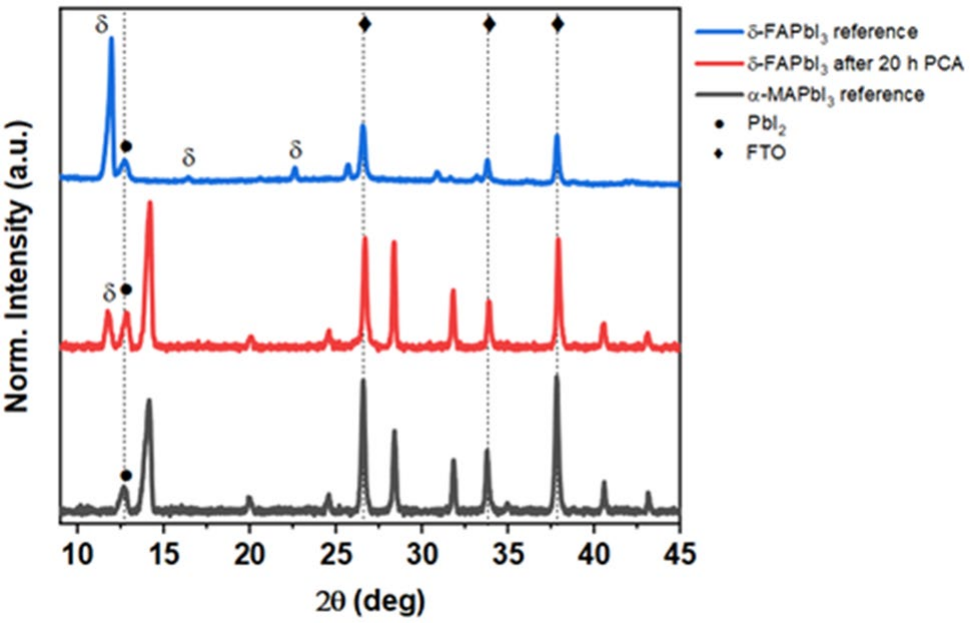
XRD pattern of the investigated δ-FAPbI_3_ sample after 20 h PCA (red) compared with reference XRD patterns (blue and black). Phase conversion of the target sample together with residual PbI_2_ impurities are observed.

The X-ray diffractograms of the α‑MAPbI_3_ thin film featured the distinctive reflexes at 14.2°, 28.4° and 31.8° corresponding to the (100), (200), (210) planes as can be seen in Fig. 3. The presence of unreacted PbI_2_ is revealed by the characteristic peak at 12.6°, which is also present in δ‑FAPbI_3_. δ‑FAPbI_3_ exhibits a characteristic intense peak at 11.9° (100). Notably, after 20 h PCA, the XRD pattern of the FAPbI_3_ thin film exhibited a minor peak at 11.9°, in addition to the peaks expected for the higher symmetry α-phase. We suggest that the migration of MA^+^ cations has a substantial impact on the stability of the mixed α-MA_x_FA_1−x_PbI_3_, which is in agreement with previous reports^23,33^. The ion motion is activated by thermal energy only, however the uptake of MA^+^ cations and subsequent incorporation into the FAPbI_3_ lattice is likely driven by the resulting stabilization. It is worth mentioning that MAPbI_3_ does not crystallize in the fashion of δ-FAPbI_3_ at low-temperature regimes. This can be attributed not only to the smaller size, insufficient to stabilize the configuration of face-sharing PbI_6_ octahedra, but also to the differences in charge distribution and prevalence of motion about different rotational axes. The MA^+^ cation preferentially aligns based on the fourfold symmetry element of the C–N axis and three-fold rotation about the C–N bond, instead for FA^+^ the prevailing axis of rotation is along the N–N direction^34–36^. Additionally, the presence of multiple cations different in size and charge distribution mitigates entropic-driven phase separation^6,37^.

**Figure 4:**
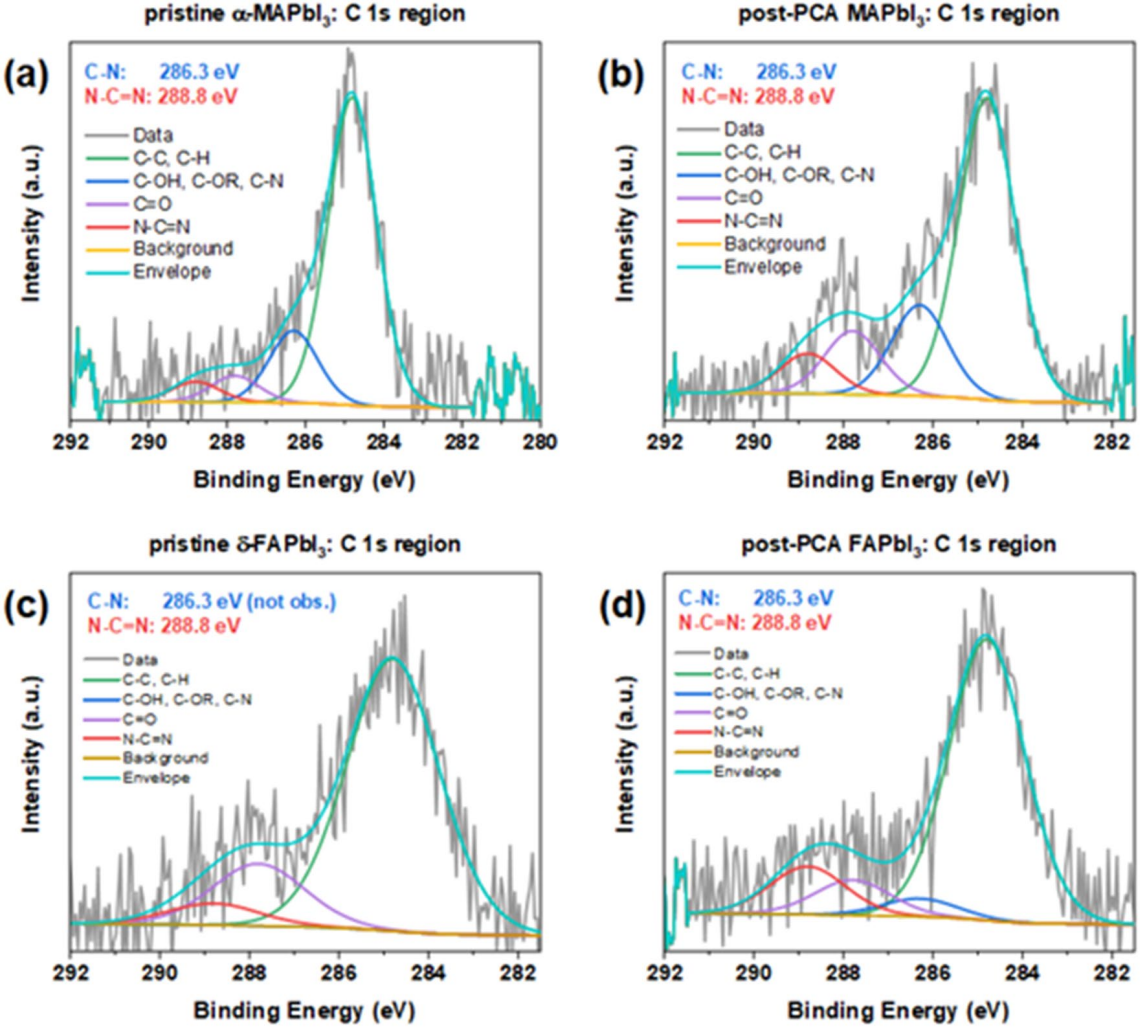
High-resolution scans of the probed C 1 s regions of (**a**) pristine α‑MAPbI_3_, (**b**) post-PCA MAPbI_3_ after 60 h annealing, (**c**) pristine δ‑FAPbI_3_, (**d**) post-PCA FAPbI_3_ after 60 h annealing.

The X-ray photoelectron spectroscopy (XPS) of pristine and annealed films confirmed the presence of MA^+^ (FA^+^) in the post-PCA FAPbI_3_ (MAPbI_3_) thin films after 60 h of annealing. The identification of the intermixing cations is possible due to the different nature of the carbon–nitrogen bonds in the two organic cations, resulting in characteristic binding energies in the C 1s and N 1s regions of the XPS spectra^38,39^. Figure 4a shows a prevalence of the single C–N bond at 286.3 eV in the pristine MAPbI_3_. The N–C=N contribution at 288.8 eV becomes pronounced in the post-PCA sample (Fig. 4b). Analogously in Fig. 4c the C–N contribution is buried under the background for the pristine FAPbI_3_, but it becomes observable post-PCA (Fig. 4d). Similar conclusions can be drawn regarding the N 1s region of the MAPbI_3_ thin films (Fig. S4). Table S1 lists the elemental concentrations of the thin films calculated via fitting of the photoelectron spectra. Although they should not be interpreted quantitatively, they indicate a decrease (increase) of the C/N ratio for post-PCA MAPbI_3_ (FAPbI_3_), due to the higher (lower) content of nitrogen atoms in FA^+^ (MA^+^).

**Figure 5:**
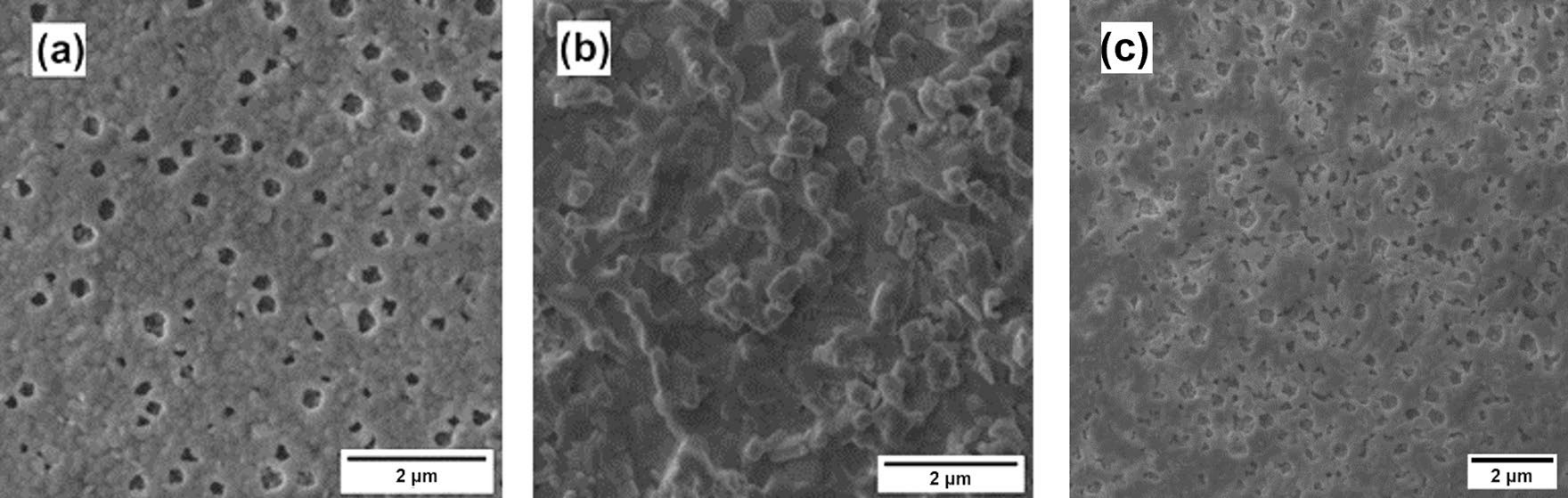
Top-view SEM images of (**a**) as-prepared α‑MAPbI_3_ thin film, (**b**) post-PCA MAPbI_3_ thin film, (**c**) α‑MAPbI_3_ thin film annealed on its own (no PCA) for 80 h.

The scanning electron microscopy (SEM) images of the as-prepared and post-PCA α‑MAPbI_3_ thin films (Fig. 5) showed that the morphology of the MAPbI_3_ is greatly affected by the PCA procedure. The abundant defects and pinholes of the as-prepared material appeared to be healed upon PCA. For the sake of comparison, an additional α‑MAPbI_3_ thin film was annealed for 80 h that resulted in the expected increase in the grain size, however the surface still displayed pinholes that became larger due to densification process. Apparently, the thermally-induced migration of FA^+^ into the MAPbI_3_ thin film (and considering that iodide ions will also migrate), resulted in mass flux responsible for the removal of pinholes. The understanding of diffusive phenomena is enshrined in Fick’s first law of diffusion, summarized by the equation J (x) = –D dφ/dx, where J is the diffusion flux, D is the diffusivity and φ is the concentration of the substance. In our system, two different-sized cations having different D coefficients diffuse in a bidirectional fashion. Using the approximations reported elsewhere^31^, we calculated the diffusion lifetime τ_d_ by fitting the absorbance data with monoexponential fits and subsequently the effective diffusivity coefficient at 100 °C by the formula D_eff_ = L^2^/τ_d_. We found τ_d_ = 1067 min^−1^ and D_eff_ = 2.50 × 10^–14^ cm^2^ s^−1^. Interestingly, the diffusion lifetime is ~ 5 times slower than the one reported by *Elmelund *et al. for the bidirectional diffusion of bromide and iodide ions in MAPbX_3_ thin films at the same temperature^31^. This is in good agreement with the previously mentioned theoretical modeling, that predicts a higher activation energy for the A-site cations compared to the halide ions. From the SEM images in Figs. 5 and 9 below, we can see that the post-PCA films show a certain porosity, absent in the as-prepared thin films and in those annealed without contact. One possible explanation to this observation is the formation of pores in connection with the Kirkendall effect^40^. The well-known Kirkendall effect describes the displacement of the interface between two distinct materials upon interdiffusion, caused by the difference in the diffusivities of each component that in turn results in different diffusion rates^41,42^. When long diffusion times are allowed, e.g. in the present work, one side will densify and the other will be richer in vacancies, giving rise to the pores. This intriguing phenomenon has been reported for MAPbBr_3_^43^, however further investigations are needed to provide conclusive elucidations.

The healing of pinholes in perovskite thin film were shown to happen with post treatments e.g. solvent-annealing with water/DMF^44^, MAI solution^45^ or MA gas treatment^46^. Therefore, a vapor-assisted healing of pinholes might be also the case in our experiments and will be addressed below.

**Figure 6:**
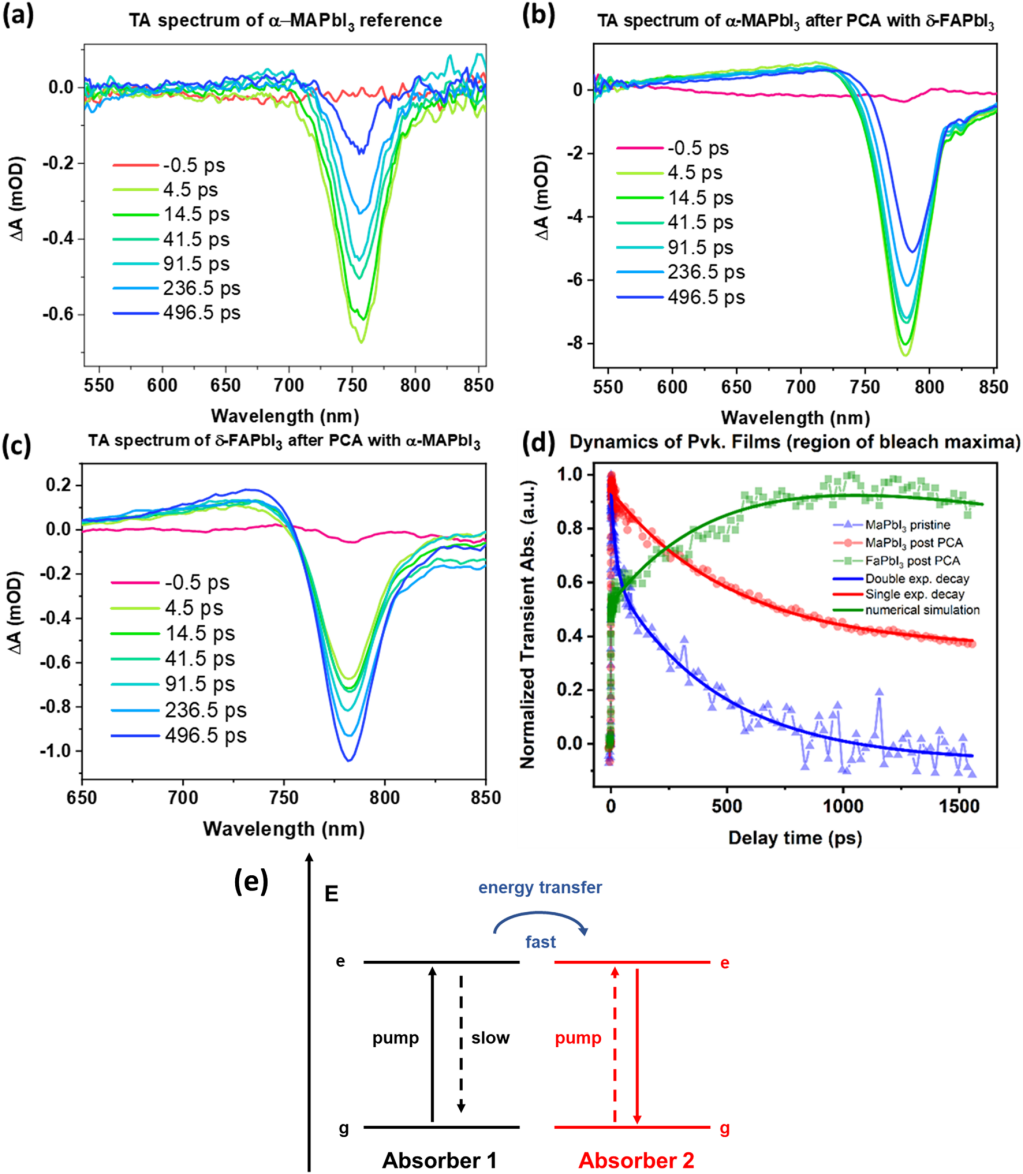
Transient absorption spectra of (**a**) pristine α‑MAPbI_3_ (**b**) post-PCA MAPbI_3_, (**c**) post-PCA FAPbI_3_. (**d**) Dynamics of the thin films around the bleaching maxima. The exponential decay fittings are shown for α‑MAPbI_3_ and post-PCA MAPbI_3_, whereas the post-PCA FAPbI_3_ data were fitted with a numerical simulation. (**e**) Hypothesized energy level diagram for the post-PCA FAPbI_3_ dynamics.

The charge and excitation dynamics of the materials and the effect of paired contact annealing were probed using ultrafast transient absorption spectroscopy (TAS). The as-prepared α‑MAPbI_3_ and post-PCA MAPbI_3_ and FAPbI_3_ after 80 h annealing were probed after a 150 fs pulse with an excitation wavelength of 400 nm. In this case, only the as-prepared MAPbI_3_ was investigated, because pristine δ-FAPbI_3_ does not show absorption edges in the probed region (550–850 nm, compare to Fig. 2d). Therefore, no relevant features can be expected in the transient spectrum. The TA spectra are shown in Fig. 6a–c. All spectra feature a negative signal around the band edge, which is assigned to photobleaching of the ground state. The wavelength of the bleaching maxima corresponds exactly to the absorption band edge and the PL emission peaks after 80 h PCA for each material (compare Fig. 2). Notably, the amplitude of the post-PCA MAPbI_3_ photobleaching is significantly larger than both the as-prepared α-MAPbI_3_ and the post-PCA FAPbI_3_, when the fluence was kept constant for all measurements. One possible reason could be the augmented response due to the pinhole and defect healing induced by PCA as previously shown in the SEM images.

More insight can be gained by investigating the dynamics of the photobleach signals (Fig. 6d). The recombination lifetime was calculated for α‑MAPbI_3_ and post‑PCA MAPbI_3_ fitting the signals with single and double exponential functions. Interestingly, a longer effective lifetime of τ_eff_ = 525 ± 22 ps is calculated for the post‑PCA MAPbI_3_, 200 ps longer than the pristine counterpart (τ_eff_ = 324 ± 45 ps). We attribute this again to the evident pinhole and defect healing of the MAPbI_3_ thin film. It is widely accepted that in organic–inorganic lead halide perovskite absorbers the presence of pinholes substantially reduces the charge carrier lifetimes, as they provide excitonic recombination paths^47,48^. Therefore, the macroscopic self-healing might be the reason for the enhanced charge carrier lifetime.

The dynamics of post-PCA FAPbI_3_ are more complex. The dynamics show a coherent response at zero time delay as well as a growing component, indicating that more than  one absorber is excited here. We assign these dynamics as follows: after rapid excitation (similarly to both MAPbI_3_ cases), a fast energy transfer happens between two absorbers, for example, MA_x_FA_1−x_PbI_3_ on the surface and FAPbI_3_ in the bulk of the analyzed substrate (Fig. 6e). The time-resolved absorption data were therefore simulated numerically using two interacting absorbers (details can be found in the SI). The growth time of the corresponding simulation is 530 ps, while the slow decay visible for high delay times is best fitted by 5500 ps. The growing time matches the decay of post-PCA MAPbI_3_, further underlining our assignments (details about data fitting are provided in Tables S2, S3 and Note 2, Supporting Information).

**Figure 7:**
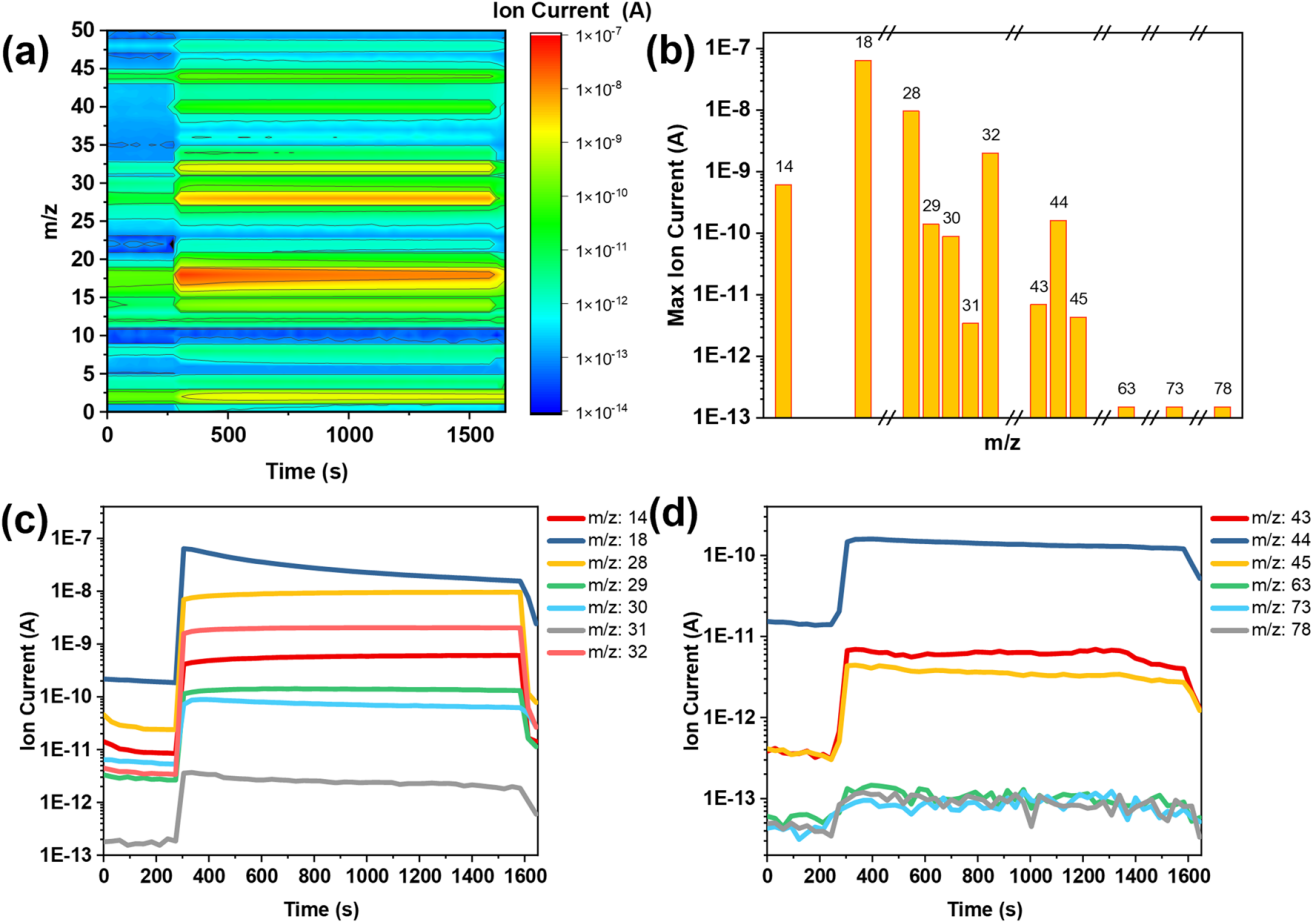
(**a**) Color-coded 3D plot showing the intensity of each m/z over time. (**b**) Histogram plot showing the absolute maximum intensity of m/z values of interest. (**c**,**d**) Intensities of selected m/z signals over the experiment time. Around t = 300 s the valve was opened leading the gas flow to the detector.

The experimental observations demonstrate the presence of the ‘foreign’ FA^+^ (MA^+^) cation on the post-PCA MAPbI_3_ (FAPbI_3_) samples, however, the origin of their presence might have different reasons. Alongside solid-to-solid ion diffusion, two other processes might be possible: (i) solvent-induced vapor transfer, implying that residual DMF and/or DMSO might dissolve the perovskite and carry its components to the other substrate through the vapor phase. (ii) Simple vapor transfer, meaning that upon heating the gaseous molecules methylamine (MA) and formamidine (FA) are formed and reach the surface of the opposite substrate. To gain a look into these processes, in-situ mass spectroscopy (MS) was used to assess the formation of small molecules when α-MAPbI_3_ and δ-FAPbI_3_ thin films on FTO were put together in a chamber and heated, hence recreating the experimental conditions. The temperature in the MS chamber was gradually raised from room temperature to 105 °C in a 20-min time interval. The color-coded 3D plot in Fig. 7a shows the detection of ionic fragments (m/z) over time and the most relevant m/z peaks are plotted in Fig. 7b. No molecular peaks are observed for the used solvents DMF and DMSO (m/z = 73 and 78 respectively), whereas the signals corresponding to the system methylammonium/methylamine (m/z = 32, 31) and formamidinium/formamidine (m/z = 45, 44) are either very low or overlap with common fragments such as O_2_^·+^ (m/z = 32) or CO_2_^·+^ (m/z = 44). Figure 7c,d show the intensities over time of m/z values of interest. Interestingly, the signals at m/z = 30 and 31 decrease over time, while for m/z = 43 an increasing trend is observed. These results are counter‑intuitive, as the methylammonium adduct is less thermally stable than its formamidinium counterpart. To sum up, the found amount of residual DMF and DMSO solvents is comparatively low. The same can be said for the MA/MA^+^ and FA/FA^+^ systems in the vapor phases, even though the overlapping O_2_, CO_2_, and DMF‑related signals hamper the analysis. Therefore, while the solvation effects can be highly likely ruled out, further studies are required to determine if the migration phenomena are purely diffusive or if the vapor generation plays a non-negligible role.

**Figure 8:**
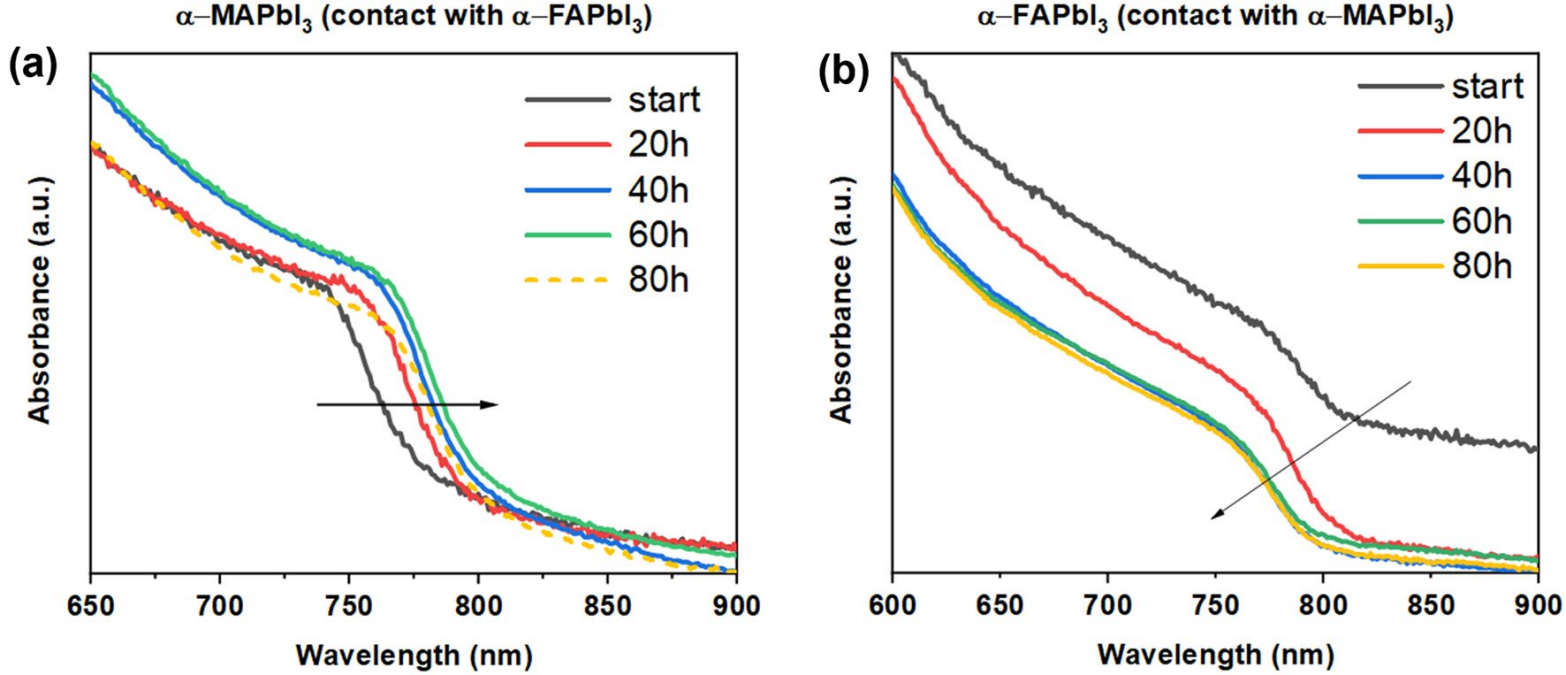
UV–vis spectra of (**a**) α-MAPbI_3_ and (**b**) α-FAPbI_3_ over 80 h physical contact annealing at 100 °C. The black line in (**b**) shows an offset due to an instrumental error.

To see if the solid-to-solid diffusion is independent from the crystal phase, we investigated a couple of α-MAPbI_3_/α-FAPbI_3_ and similarly the UV‑vis spectra were recorded every 20 h, in which a red‑shift of the absorption edge from 791 to 810 nm in α-MAPbI_3_ and a blue‑shift from 875 to 801 nm in α-FAPbI_3_ was observed as shown in Fig. 8. *Tauc* plots were constructed from these spectra to estimate the band gap of the materials and their change over annealing time (Fig. S2).

**Figure 9:**
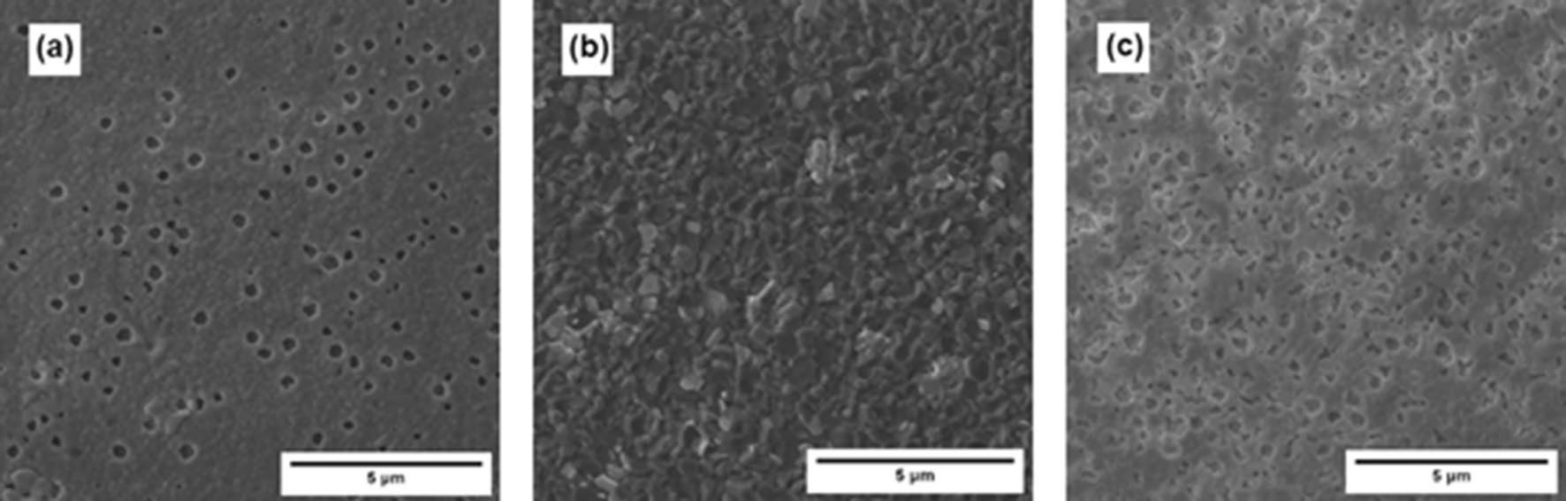
Top-view SEM images of (**a**) as-prepared α‑MAPbI_3_, (**b**) post-PCA MAPbI_3_ (**c**) post-annealing (non-PCA) MAPbI_3_. Upon simple annealing there is an increase in the grain size, but complete defect healing is observed only upon PCA. The same annealing time was used for (**b**,**c**), i.e. 80 h.

Interestingly, the estimated band gap of the α‑MAPbI_3_ layer shifted from 1.59 to 1.55 eV. The decrease of the band gap could be a hint of the diffusion of FA^+^ ions into the α‑MAPbI_3_ thin film, yielding a modified composition MA_1−x_FA_x_PbI_3_. The defect-healing effect on the morphology previously discussed for the α/δ case could be observed for the α-MAPbI_3_/α-FAPbI_3_ as well, as shown in the SEM images of the thin films in Fig. 9.

The observations manifested that the solid-to solid diffusion of A-site cations is independent from the crystalline phase, however it was more obvious in the black α-MAPbI_3_/yellow δ-FAPbI_3_ couple due to the diffusion induced phase transition.

## Conclusion

The physical contact annealing (PCA) of APbI_3_ (A = MA^+^, FA^+^) showed an impact on the physical properties of the perovskite thin films, likely due to the slow and mutual migration of MA^+^ and FA^+^ into the opposite layer. For the α-MAPbI_3_/δ-FAPbI_3_ couple, a visually observable yellow to brown color change in the original δ-FAPbI_3_ thin film indicated a phase and composition change presumably triggered by the stabilizing effect of migrated MA^+^ cations. Additionally, the pinholes and voids present in the original α-MAPbI_3_ thin films were evidently healed upon PCA, resulting in longer lifetimes of the charge carriers. X-ray photoelectron spectroscopy has further demonstrated the presence of the ‘foreign’ cation on the post-PCA thin films. For α-MAPbI_3_/α-FAPbI_3_ similar observations were made, as the estimated band gap also shifted upon PCA. These findings pave an insight into the flexibility of the hybrid perovskite lattice and the cation dynamics within the perovskite solid phases. Ion migration could be detrimental for device application and a deeper understanding of factors enhancing cationic drifts and procedures to circumvent the ion movements are crucial to optimize device architectures and compositional engineering of perovskites. The mechanism of the migration process and controlling parameters are part of our further investigations, in particular the question if we can completely rule out a vapor-assisted diffusion, whereas solvent-induced effects can be ruled out, possibly indicating a mainly diffusive pathway.

## Materials and methods

### Chemicals

FTO substrates were from *Sigma Aldrich* (thickness 2 mm, ~ 7Ω/sq), PbI_2_ (99.99%, trace metals basis) from *TCI Chemicals,* methylammonium iodide (> 99.99%) and formamidinium iodide (> 99%) were purchased from *Greatcell Solar*. Toluene, dimethylformamide (99.8%, extra dry) and dimethyl sulfoxide (99.7 + %, extra dry) were purchased from *Acros Organics.*

### Handling of FTO substrates

All thin films were fabricated on self‑cut FTO substrates of size 1.5 × 2.0 cm or 1.5 × 1.5 cm. The substrates were cleaned via sonication in *Hellmanex III* (2% v/v solution in deionized water), deionized water and absolute ethanol, in that order, for 20 min each. The substrates were dried using pressurized air and stored carefully. Before the film deposition, the substrates were treated with UV/ozone for 15 min.

### Fabrication of α‑MAPbI_3_thin films

The MAPbI_3_ precursor solutions were prepared by dissolving PbI_2_ and MAI in a 1:1 molar ratio in DMF, to yield a 1 M solution. 100 μL of precursor solution were deposited on the clean FTO/glass substrate via a micropipette and the solution was spin-coated at 4000 rpm (4000 rpm/s) for 30 s. After 20 s from the start of the deposition, 120 μL of toluene (anti-solvent) were dropped on the spinning substrate. After spin coating, the substrates were heated on a hot plate at 100 °C for 30 min to enhance crystallization and remove residual organic solvents.

### Fabrication of δ‑FAPbI_3_thin films

The FAPbI_3_ precursor solutions were prepared by dissolving PbI_2_ and FAI in a 1:1 molar ratio in a DMF/DMSO 4/1 v/v mixture, to yield a 1 M solution. 100 μL of precursor solution were deposited on the clean FTO/glass substrate via a micropipette and the solution was spin coated at 4000 rpm (4000 rpm/s) for 30 s. After spin coating, the substrates were heated on a hot plate at 100 °C for 30 min to enhance crystallization and remove residual organic solvents.

### Physical contact annealing

After fabrication, the thin films were physically paired with the coated surfaces facing each other and they were clamped together using office paper clips. The paired substrates were put in a furnace in the air at 100 °C.

### Characterization

The UV–visible absorption spectra of the perovskite layers were measured in a *Lambda 950* UV‑vis spectrometer from *PerkinElmer*. The photoluminescence spectroscopy measurements were conducted using a fluorescence spectrometer model *LS-55* from *PerkinElmer*. The thin films were excited at 600 nm with a narrow band of a few nanometers. The X‑ray diffraction patterns of the perovskite layers were measured in a *STADI MP* X-ray diffractometer from *STOE* with Cu-K_α1_ radiation (λ = 1.5406 Å) operating in reflection mode. XPS measurements were performed on an ESCA M-Probe spectrometer from *Surface Science Instruments* under reduced pressure of 10^–9^ mbar using a monochromatic Al Kα (1486.6 eV) radiation. Spectral corrections to the C1s signal (284.8 eV) and compositional calculations were carried out via the software *CasaXPS*. Peak fitting of the raw data was performed with Gaussian–Lorentzian functions GL(30) and a Shirley background. The thin films’ surfaces were studied using a focused ion beam scanning electron microscope (FIB-SEM) *Strata Dual Beam 235* from *FEI*. Transient absorption measurements: for femtosecond pulse pump white light probe experiments at 125 kHz, a *PHAROS* Yb:KGW‑based laser system equipped with regenerative amplifier was used. A non‑collinear optical parametric amplifier (NOPA) from the company *Light Conversion* was used for pump, allowing tuning of the wavelengths from near UV to near IR. The temporal resolution is 100–150 fs for the laser as well as for the NOPA system. The output of the amplifier was attenuated using appropriate band pass filters. The attenuated pulse was focused onto the sample with a spot diameter of ca. 100 µm. As regards the probe, a laser beam with wavelength 800 nm was focused onto a 2 mm thick sapphire plate to yield a white light super continuum. The beam was filtered into the intended range of 480–900 nm. For the detection of the TA spectra, a silicon‑based diode array mounted in a spectrometer *HARPIA* from *Light Conversion*. The undesirable background noise was blocked setting the polarization of the excitation beam perpendicular to that of the probe light and positioning a polarizer in front of the detector. The MS experiment was carried out using a quadrupole *QMS 220 M3* mass spectrometer from *Pfeiffer*. 70 V electron ionization from yttrium coated iridium filaments was employed. A secondary electron multiplier with a potential of 940 V was used. The filter time was set to 100 ms/amu.

## Supplementary Information

Supplementary Information.

## Data Availability

All data generated or analysed during this study are included in this published article and its supplementary information files.
