# Supplementary material for: Thermally-induced drift of A-site cations at solid–solid interface in physically paired lead halide perovskites
Source: Sci Rep. 2022 Jun 17;12:10241. doi: 10.1038/s41598-022-14452-y (PMC9205985; doi:10.1038/s41598-022-14452-y)
Supplement: Supplementary file 1 — Supplementary Information. [file 41598_2022_14452_MOESM1_ESM.docx]

**Supporting Information**

# Thermally-induced drift of A-site cations at solid-solid interface in physically paired lead halide perovskites

Daniele T. Cuzzupè^1,2,a^, Feray Ünlü^1,a^, Khan Lê^1^, Robin Bernhardt^3^, Michael Wilhelm^1^, Matthias Grosch^1^, Rene Weißing^1^, Thomas Fischer^1^, Paul H. M. van Loosdrecht^3^, Sanjay Mathur^1^*

^a^ D.T.C. and F.Ü. contributed equally to this work

^1^Institute of Inorganic Chemistry, Chemistry Department, University of Cologne, Greinstr. 6, 50939, Cologne

^2^Current affiliation: Department of Physics, University of Konstanz, D-78457 Konstanz, Germany

^3^ Institute of Physics 2, University of Cologne, Zülpicher Str. 77, 50937 Cologne, Germany

*Corresponding email: [sanjay.mathur@uni-koeln.de](mailto:sanjay.mathur@uni-koeln.de)


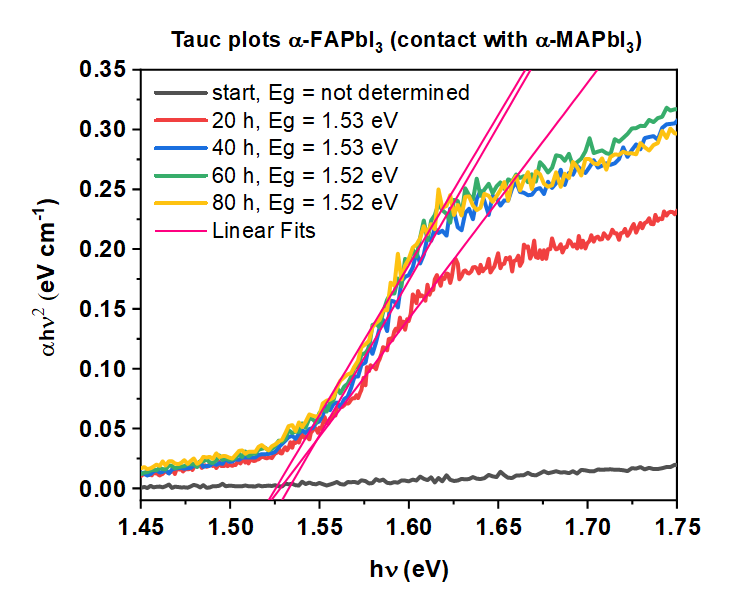

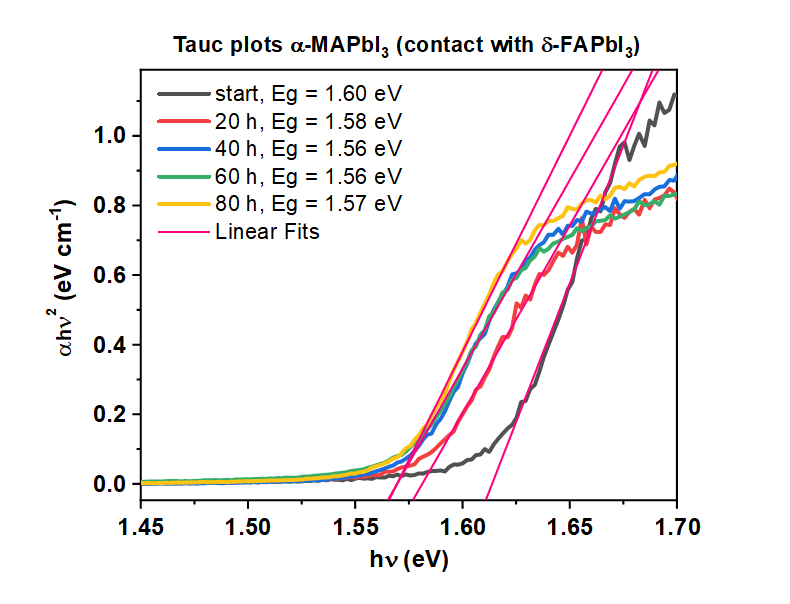


a) b)

**Figure S1:** Tauc plots of (a) α-MAPbI_3_ thin film over 80 h PCA with α-FAPbI_3_ (b) α-FAPbI_3_ thin films in contact with α-MAPbI_3_. The estimated bandgap value for each plot is shown in the legend.


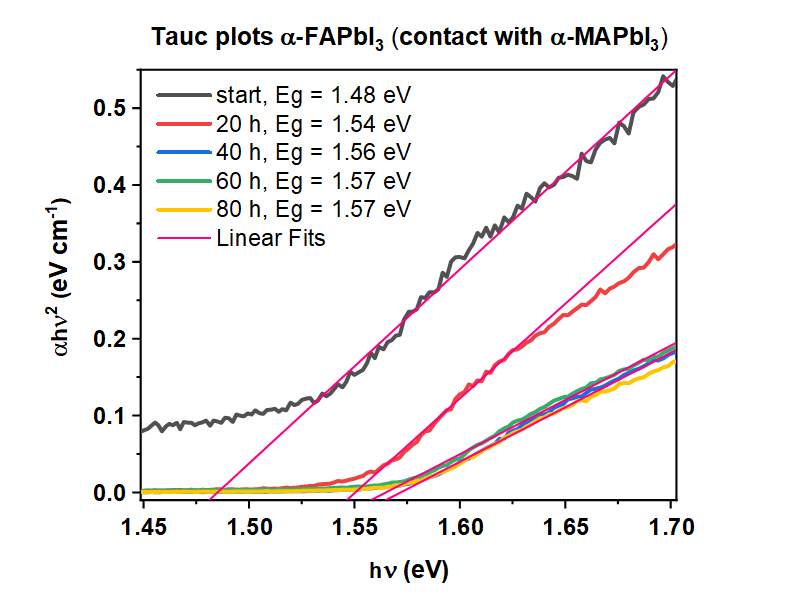


a) b)

**Figure S2:** Tauc plots of (a) α-MAPbI_3_ thin film over 80 h PCA with α-FAPbI_3_ (b) α-FAPbI_3_ thin films in contact with α-MAPbI_3_. The estimated bandgap value for each plot is shown in the legend.

**Figure S3:** Plots used to estimate the Urbach energy of an α-MAPbI_3_ thin film over 80 h PCA with α‑FAPbI_3_. The estimated Urbach energy for each plot is shown in the legend.

**Note 1:** Calculation of the estimated Urbach energy (*E*_U_)

To estimate the Urbach energy, we first need to estimate the absorption coefficient. For diluted solutions, it is possible to determine the absorption coefficient from the absorbance data by the Lambert-Beer’s law. For thin films there are interference effects that limit the applicability of this law, and the thickness of the thin film does not correlate linearly with the absorbance anymore. However, in the absence of more accurate data on the absorption coefficients that require specific experimental setups, the Lambert-Beer’s law has been widely used empirically in the practice, to provide qualitative information.

Given the Lambert-Beer’s law in the form:

(1) $I \left( z \right)=I_{0}\exp\left( -\alpha z \right)$

and considering the beam path z equal to the thickness of the thin film *d*, we can rearrange equation (1) as follows:

(2) $\ln\left( \frac{I_{0}}{I\left( z \right)} \right)= \alpha d$

To which follows, applying the relation between absorbance and transmittance and the conversion from natural logarithm to logarithm in base 10:

(3) $\alpha=\frac{2.303 A}{d}$ ,

where A is the measured absorbance expressed in arbitrary units and d is the thickness of the material.

Since *d* is assumed constant for a specific thin film, and in our case *d* = 400 nm, in this approximation the absorption coefficient is a scale of the absorbance data.

The Urbach energy is related to the absorption coefficient via the equation:

(4) $\alpha=\alpha_{0}\exp\left( \frac{h\nu- E_{c}}{E_{U}} \right)$,

where α_0_ and *E*_c_ are material constants. Following equation (4), ln (α) as determined in equation (3) is plotted against the photon energy hν. A linear fit of the linear part of the obtained plots allows to extract *E*_U_ as the reciprocal of the slope of the fitted line (**Figure S3**).

**Table S1**: Computed elemental concentration of the pristine and post-PCA MAPbI_3_ and FAPbI_3_ thin films retrieved by fitting of the XPS spectra. The small Sn and O concentrations are attributed to underlying FTO.

| Element | pristine α‑MAPbI_3_ (at. %) | post‑PCA MAPbI_3_  (at. %) | pristine  δ‑FAPbI_3_  (at. %) | post‑PCA δ‑FAPbI_3_  (at. %) |
| --- | --- | --- | --- | --- |
| C 1s | 35.56 | 24.54 | 23.04 | 25.46 |
| N 1s | 3.80 | 13.51 | 6.55 | 3.99 |
| Pb 4f | 26.89 | 17.93 | 17.11 | 23.10 |
| I 3d | 28.31 | 40.35 | 20.56 | 19.80 |
| (Sn 3d) | 1.47 | 0.21 | 5.88 | 6.50 |
| (O 1s) | 3.96 | 3.46 | 24.23 | 21.15 |
| (Cl 2p) | - | - | 2.63 | - |


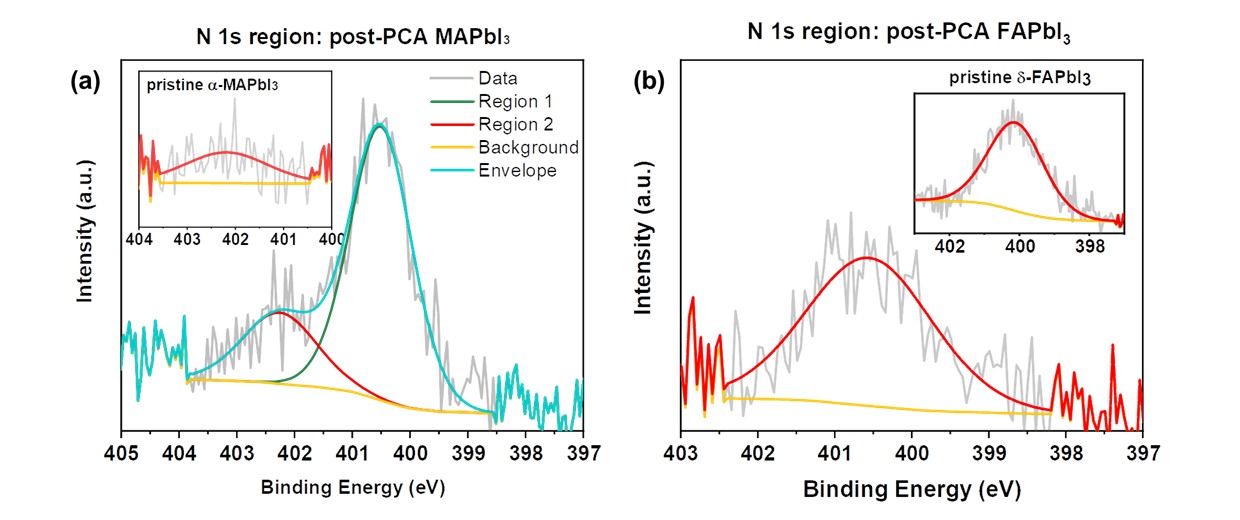


**Figure S4:** High resolution scans of the probed N 1s regions of (a) post-PCA MAPbI_3_, inset: pristine α‑MAPbI_3_ (b) post-PCA FAPbI_3_, inset: pristine δ‑FAPbI_3_.

**
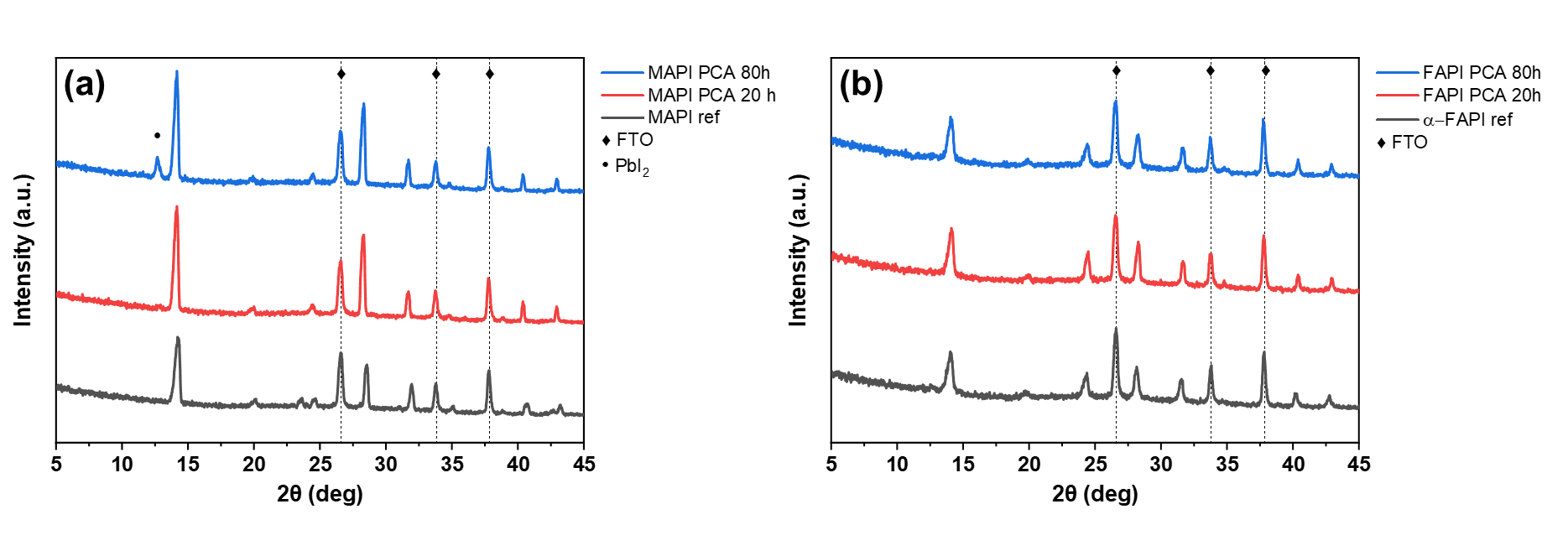
**

**
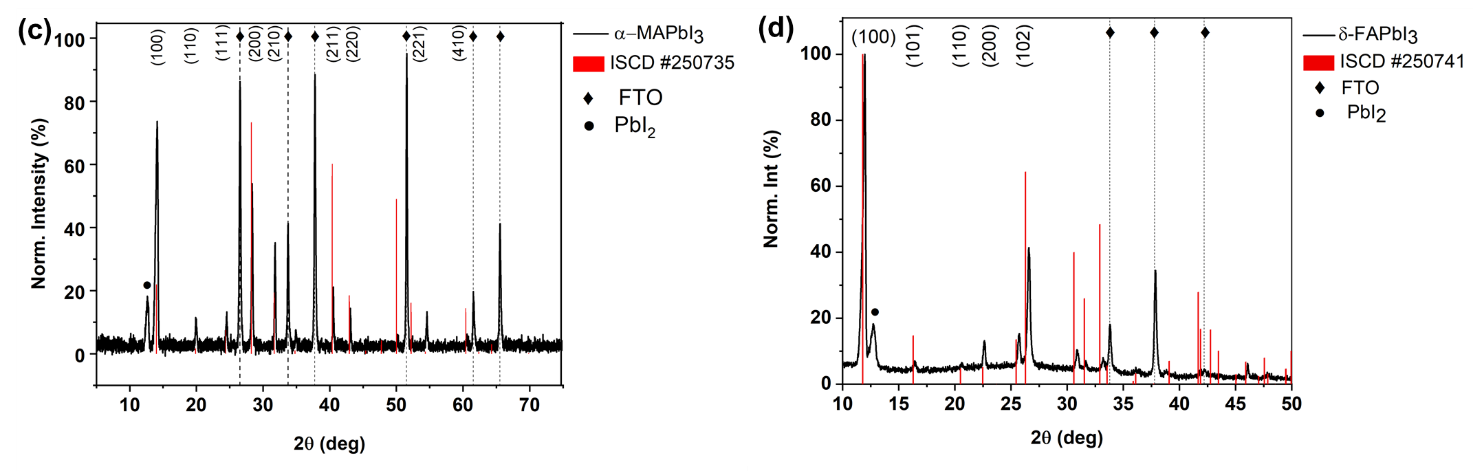
**

**Figure S5**: XRD patterns of a) α-MAPbI_3_ thin film as-prepared and after 20 h, 80 h PCA and
b) α-FAPbI_3_ thin film as-prepared and after 20 h, 80 h PCA; XRD patterns compared with reference diffractograms from *Stoumpos et al.*^[1]^ for c) MAPbI_3_ and d) FAPbI_3._

**Figure S6:** Dynamics of the pristine and post-PCA MAPbI_3_ thin films in the first ~1600 ps after excitation, evaluated in the region of the photobleaching maximum. The decays are fitted with decay functions (see Table S2-S3).


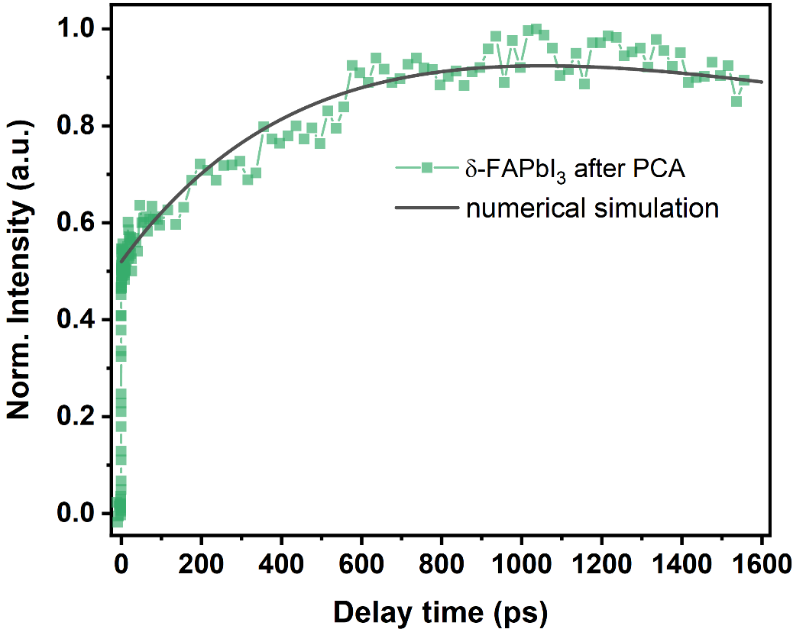


**Figure S7**. Dynamics of the post-PCA FAPbI_3_ thin film in the first ~1600 ps after excitation, evaluated in the region of the photobleaching maximum. The inset lists the three custom functions used for the fitting of the transient data.

**Tables S2-S3:** Functions used for the fitting of the transient absorption decay data and obtained fitting parameters.

| **Model** | Double Exponential Decay (Offset) |
| --- | --- |
| **Equation** | y = y_0_ + A_1_ exp (-x/t_1_) + A_2_ exp (-x/t_2_) |
| **Dataset** | pristine α-MAPbI_3_ |
| **y_0_** | -0.06754 ± 0.01856 |
| **A_1_** | 0.32068 ± 0.02827 |
| **t_1_** | 21.80272 ± 4.33431 ps |
| **A_2_** | 0.69225 ± 0.02384 |
| **t_2_** | 458.55594 ± 46.87283 ps |
| **t_avg_** | 320 ± 45 ps |

| **Model** | Single Exponential Decay (Offset) |
| --- | --- |
| **Equation** | y = y_0_ + A_1_ exp (-x/t_1_) |
| **Dataset** | post-PCA MAPbI_3_ |
| **y_0_** | 0.354 ± 0.009 |
| **A_1_** | 0.587 ± 0.009 |
| **t_1_** | 525.26 ± 22.14 ps |

**Note 2:** Numerical simulation for the fitting of the post-PCA FAPbI_3_ dynamics

Considering two coupled systems as shown in figure 6e, the dynamics of the populations n_1_ and n_2_ can be described using a coupled rate model.

$$\frac{\partial n_{1}}{\partial t}= -k_{10}n_{1}-k_{12}n_{1}+k_{21}n_{2}; \frac{\partial n_{2}}{\partial t}= -k_{20}n_{2}-k_{21}n_{2}+k_{12}n_{1}$$

To limit the amount of free parameters, we consider a few simplifications:

1. n_2_ effectively only transfers to n_1_ and not to its ground state 🡪 k_20_ = 0
2. n_1_ does not transfer to n_2_ 🡪 k_12_ = 0

The model now simplifies to:

$$\frac{\partial n_{1}}{\partial t}= -k_{10}n_{1}+k_{21}n_{2}; \frac{\partial n_{2}}{\partial t}= -k_{21}n_{2}$$

This formula only leaves two free parameters, k_21_ and k_10_.

To account for the initial response of the system at t=0, we need to assume that both populations have been excited. Therefore, we have an initial population N_1_ = n_1_(t=0) and N_2_ = n_2_(t=0). Those four parameters represent our fit parameters.

The fit can be seen in the figure below. The best fitting simulation with respect to the normalized dynamics spectrum yields:

| N_1_ | 0.52 (a.u.) |
| --- | --- |
| N_2_ | 0.65 (a.u.) |
| k_21_ | 530 ps |
| k_10_ | 5500 ps |

**Figure S7:** Data used for the calculation of the effective diffusion rate starting from absorbance data. The fitting equation and the extracted fitting parameters are displayed in the inset.

[1] C. C. Stoumpos, C. D. Malliakas, M. G. Kanatzidis, *Inorg. Chem.* **2013**, *52*, 9019–9038.
